# Distinct late Pleistocene subtropical-tropical divergence revealed by fifteen low-copy nuclear genes in a dominant species in South-East China

**DOI:** 10.1038/s41598-021-83473-w

**Published:** 2021-02-18

**Authors:** Jun-Wei Ye, De-Zhu Li

**Affiliations:** 1 Germplasm Bank of Wild Species in Southwest China, Kunming Institute of Botany, Chinese Academy of Sciences, Kunming, 650201 Yunnan China; 2 Natural History Research Centre of Shanghai Natural History Museum, Shanghai Science & Technology Museum, Shanghai, 200041 China

**Keywords:** Haplotypes, Population genetics, Molecular ecology, Population dynamics, Population genetics

## Abstract

In East Asia, genetic divergence is usually considered to be correlated to different floristic regions, however, subtropical-tropical divergence is largely ignored, compared to widely explored temperate-subtropical divergence. *Lindera aggregata* (Lauraceae), a dominant species in South-East China was selected to address this issue. Fifteen low-copy nuclear genes (LCGs) and four chloroplast DNA (cpDNA) fragments were used to detect its evolutionary history. In LCGs, STRUCTURE and dated Bayesian phylogeny analyses detect distinct subtropical-tropical divergence since late Pleistocene. Approximate Bayesian calculation (ABC) further supports the distinct subtropical-tropical divergence, and close related Taiwan and South China populations are diverged at the last interglacial. Isolation by distance, isolation by environment and isolation by resistance analyses suggest the current climatic difference rather than geographical distance contributes to the genetic differentiation. Principle component analysis shows populations of tropical cluster occur in warmer area with higher precipitation. Ancestral area reconstruction based on Bayesian phylogeny indicates that ancestral *L. aggregata* populations are distributed in tropical region. In cpDNA, although unique haplotypes are found in tropical region, distinct subtropical-tropical divergence is absent. In conclusion, distinct late Pleistocene subtropical-tropical divergence of *L. aggregata* is triggered by climate. It is likely that *L. aggregata* is originated in Southwest-South China and experienced hierarchical dispersal from south to north. The South China Sea land bridge has dual role in connecting or isolating Taiwan and mainland populations since the last glaciation.

## Introduction

East Asia harbours a great biodiversity in Northern Hemisphere because of extreme geo-diversity with conjunction of historical climate and sea level changes^1^. Floristic region, defined as the assemblage of all plants in a given region and time, is formed through tempo-spatial evolution and distribution of plants under historical and contemporary geo-climate conditions^2^. Different floristic regions are usually relate to different geologic structures, geomorphic units or climatic zones^2^. Different floristic regions in temperate, subtropical and tropical climates from north to south in East Asia have uneven biodiversity^3^ and likely distinct evolutionary histories^4^.

Genetic divergence corresponding to temperate-subtropical differentiation has been widely explored. The temperate-subtropical divergence was firstly found in *Acer mono* (Sapindaceae), the northern temperate populations were formed through range expansion recently while the southern subtropical populations were long-term persisted, and intermediate area in North China was occupied with admixed populations^5,6^. Similar genetic pattern was revealed in *Juglans* ssp. (Juglandaceae) and contribution of temperate-subtropical environmental difference to genetic divergence was further emphasized^7^, as different climate may has resulted in local adaptation after long-term independent evolution since late Miocene. Sharper divergence without genetic mixture was found in *Lindera obtusiloba* (Lauraceae)^8^ and *Euptelea* (Eupteleaceae)^9^. These species are more arid-sensitive and the inland Taihang-Qingling migration route in North China is not available^8,9^. Since no suitable habitat in the East China Sea (ECS) shelf was predicted during the last glacial maximum (LGM) using ecological niche modeling (ENM)^8,9^, the ECS acts as geographical barrier for genetic interchanges in *L. obtusiloba* (Lauraceae)^8^, *Euptelea* (Eupteleaceae)^9^ and some other species^10^. So, contemporarily climatic and topographical condition are both responsible for the temperate-subtropical genetic divergence.

Different floristic division also occurs in subtropical (Sino-Japanese Floristic Region) and tropical (Paleotropic Floristic Region) regions^2^, while subtropical-tropical differentiation related genetic divergence was poorly understood. Although numerous phylogeograhic studies were conducted in subtropical region, limited species stretches its distribution to the tropical region^11^. Shared chloroplast genetic components without clear subtropical-tropical divergence were widely found^13–15^. In *Tetrastigma hemsleyanum* (Vitaceae), Wang et al.^12^ found early divergence in chloroplast genome (late Pliocene) while recently genetic mixture in nuclear genome between subtropical and tropical populations. The failure to uncover subtropical-tropical divergence may due to two reasons. The first reason may relate to the real absence of subtropical-tropical divergence as the only differences are weak climatic difference and topographic barrier^2^, and the boundary of subtropical-tropical climatic zones^16^ is further south compared to that of the two floristic regions^2^. The second reason relates to methodological issues. Chloroplast genome with low mutation rate is prone to trace early demographic history before the Pleistocene, while recent divergence and admixture since the Pleistocene should turn to nuclear genome^17^, which is very limited in previous studies^12^. So, whether genetic divergence occurs between subtropical and tropical regions remains to be tested.

**Figure 1:**
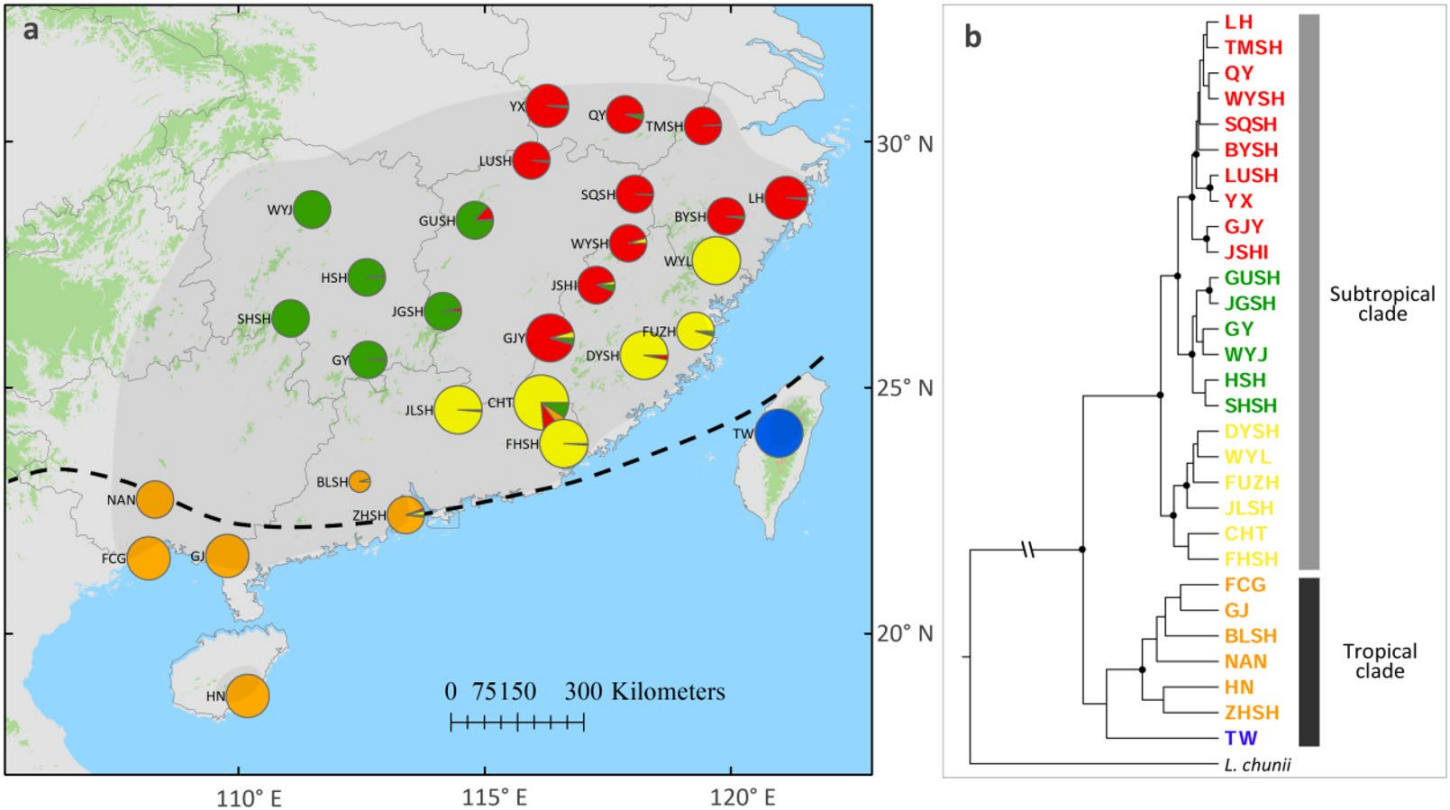
(**a**) Colour-coded grouping of the 29 *Lindera aggregata* populations according to STRUCTURE with the most likely group number *K* = 5 (ArcGis 10.2, ESRI, www.esri.com), the dashed black line represents the boundary of subtropical (Sino-Japanese Floristic Region) and tropical (Paleotropic Floristic Region) regions. The shaded area indicates the species’ distribution range. (**b**) BEAST-derived phylogeny for the 29 populations. The colour of populations indicates their genetic cluster as inferred by STRUCTURE. Statistically significant posterior probabilities (PP > 0.95) are labeled in black dots.

*Lindera aggregata* (Lauraceae) is a dominant shrub or small tree of evergreen broadleaved subtropical forests that is widely distributed in subtropical region but it can stretch to the tropical region as well (Fig. 1a)^18^. It is dioecious, produces entomophilous flowers and fleshy drupes that are putatively dispersed by birds^19^. Based on *Flora of China* (http://www.efloras.org/), there are two accepted infraspecific taxa, *L. aggregata* var. *aggregata*, and *L. aggregata* var. *playfairii* which is only distributed in South China. The difference between the two varieties lies in type of pubescence, and size of leaf and flower. Our previous phylogeographic work using four chloroplast DNA (cpDNA) fragments, *rpl16*, *psbA–trnH*, *trnL–trnF* and *trnS–trnG*, 15 self-developed low-copy nuclear genes (LCGs) and ENM uncovered distinct south-north divergence. The northern populations experienced extensive post-glacial range expansion that contrasts with its generalized long-term population stability in the southernmost range, and noteworthy post-glacial gene flow into long-term refugial populations was also found^20^.

South-north divergence accompanied with environmental difference in *L. aggregata*^20^ suggests that it is possible to detect potential subtropical-tropical divergence. However, the sampled populations were biased in subtropical region in our previous work^20^. Populations in tropical region and contact zone between south-north clusters are limited. To resolve this problem, more populations in these two regions were sampled (Table 1) and the same genetic markers were applied^20^. Bayesian phylogeny and clustering, and approximate Bayesian calculation (ABC) was conducted to detect if there is any subtropical-tropical divergence. Ancestral areas were reconstructed to infer distributions of ancestral populations. Isolation by distance (IBD)^21^, isolation by environment (IBE)^22^ and isolation by resistance (IBR)^23^ were calculated to infer driving force for genetic differentiation.

**Table 1:** Population information and chloroplast DNA haplotype distributions.

Region/Pop	Location	Lat	Long	Hap	n1/n2
**Subtropical**					
BYSH	Baiyun Mt., Zhejiang	28.48	119.90	H2 (1), H3 (7)	8/6
CHT	Changtan, Guangdong	24.70	116.15	H4 (14)	14/13
**DYSH**	Daiyun Mt., Fujian	25.66	118.24	H3 (10)	10/10
**FHSH**	Fenghuang Mt., Guangdong	23.86	116.61	H3 (5), **H5 (1), H6 (4)**	10/10
FUZH	Fuzhou, Fujian	26.15	119.28	H7 (8)	8/6
**GJY**	Ganjiangyuan, Jiangxi	26.01	116.33	H3 (8), **H8 (2)**	10/10
GUSH	Guanshan, Jiangxi	28.40	114.80	H3 (7), H9 (1)	8/6
GY	Guiyang, Hunan	25.57	112.63	H3 (8)	8/6
HSH	Hengshan, Hunan	27.24	112.60	H3 (8)	8/6
JGSH	Jinggangshan, Jiangxi	26.55	114.15	H9 (8)	8/6
**JLSH**	Jiulian Mt., Guangdong	24.55	114.46	H3 (10)	10/10
JSHI	Jiangshi, Fujian	27.08	117.27	H3 (3), H10 (5)	8/6
**LH**	Linhai, Zhejiang	28.86	121.13	H3 (8)	8/8
LUSH	Lu Mt. Jiangxi	29.62	115.95	H3 (8)	8/6
QY	Qingyang, Anhui	30.55	117.85	H3 (5), H12 (3)	8/6
SHSH	Shunhuang Mt., Hunan	26.40	111.06	H3 (6), H13 (2)	8/6
SQSH	Sanqing Mt., Jiangxi	28.93	118.05	H3 (8)	8/6
TMSH	Tianmu Mt., Zhejiang	30.32	119.43	H3 (8)	8/6
WGSH	Wugong Mt., Jiangxi	27.58	114.26	H3 (1)	1/-
WYJ	Wuyunjie, Hunan	28.62	111.49	H3 (8)	8/6
**WYL**	Wuyanling, Zhejiang	27.58	119.71	H3 (10)	10/10
WYSH	Wuyi Mt., Jiangxi	27.93	117.91	H3 (8)	8/6
**YX**	Yuexi, Anhui	30.72	116.27	H3 (8)	8/8
**Tropical**					
BLSH	Beiling Mt., Guangdong	23.09	112.46	H1 (2)	2/2
**FCG**	Fangchenggang, Guangxi	21.53	108.17	H3 (8)	8/8
**GJ**	Gaoqiao, Guangxi	21.58	109.77	H1 (6), H3 (2)	8/8
**HN**	Hainan	18.73	110.18	H3 (6), **H16 (2)**	8/8
NAN	Nanning, Guangxi	22.73	108.30	H11 (8)	8/6
**TW**	Nantou, Taiwan	24.07	120.98	**H14 (9), H15 (1)**	10/10
ZHSH	Zhongshan, Guangdong	22.41	113.40	H3 (8)	8/6

Newly added populations and derived haplotypes are labeled in bold. n1/n2 represent sampling number of chloroplast DNA and low-copy nuclear genes (LCGs), respectively.

*Pop* populations, *Lat* latitude, *Long* longitude, *Hap* chloroplast DNA haplotype.

The specific aims of the present study are to detect (1) whether subtropical-tropical divergence exists in *L. aggregata*, (2) if yes, whether geographical or climatic condition contributes to the genetic differentiation, and (3) the demographic history of *L. aggregata*.

## Results

### Haplotype distributions and Bayesian inference of cpDNA

The combined four cpDNA fragments revealed ten haplotypes and one haplotype (H3) was widely distributed in our previous work^20^. In the present study, six additional haplotypes are obtained through six additional substitution sites (Table 1 and Supplementary Table S1). The haplotype H3 is also widely distributed and more diverse haplotypes are also found in southern region (Supplementary Fig. S1). All the six new haplotypes are private in the added populations. Especially, Taiwan population is composed of two private haplotypes (H14 and H15, Supplementary Fig. S1). Although low lineage divergence is also found in Bayesian phylogeny^20^, haplotypes from Taiwan (H14 and H15) and haplotypes from populations in South China (H11 in NAN and H1 in BLSH) form a monophyletic lineage (Supplementary Fig. S1).

### Genetic structure and Bayesian inference of LCGs

In our previous study^20^, eighteen sampled populations were divided into three distinct clusters with one located in south, the second in the north-eastern part and the third in the north-western part using LCGs. In the present study, *K* = 5 is the most possible number of genetic clusters using 29 populations based on Ln*P*(*D*) and Δ*K* (Supplementary Fig. S3). The north-western and north-eastern clusters remain the same, while additional populations in the admixture and tropical region form other three clusters, which are southern cluster (in brown) and Taiwan cluster (in blue) in tropical region and north-central cluster (in yellow) located in the middle region (Fig. 1a). In the Bayesian phylogeny, the subtropical-tropical divergence is supported although the lineage of Taiwan and southern cluster has low posteriors probability supports (Fig. 1b). The divergence time of subtropical-tropical populations is estimated as 109.7 thousand years ago (ka) with 95% highest posterior density interval (HPD) as 72.9 to 171.9 ka and Taiwan-South China populations as 90.7 ka (95% HPD, 66.5–119.2 ka). High genetic differentiation is found between tropical and subtropical clusters with the highest found between Taiwan and other clusters (Table 2). Taiwan cluster has low genetic diversity, especially haplotype richness, *A*_R_, while the other four clusters have similar level of genetic diversity (Table 2).

**Table 2:** Pairwised genetic differentiation (*F*_ST_) and genetic diversity as measured by π and haplotype richness, *A*_R_, among the five potential clusters as inferred by STRUCTURE in low-copy nuclear genes (LCGs).

	North-eastern	North-western	North-central	Southern	Taiwan	*A*_R_	π (× 10^−3^)
North-eastern	0.00					3.74	2.84
North-western	0.09	0.00				3.68	2.61
North-central	0.14	0.14	0.00			4.71	3.18
Southern	0.39	0.37	0.21	0.00		4.22	3.15
Taiwan	0.57	0.49	0.46	0.45	0.00	1.70	2.03

### DIYABC analyses and ancestral area reconstructions

**Figure 2:**
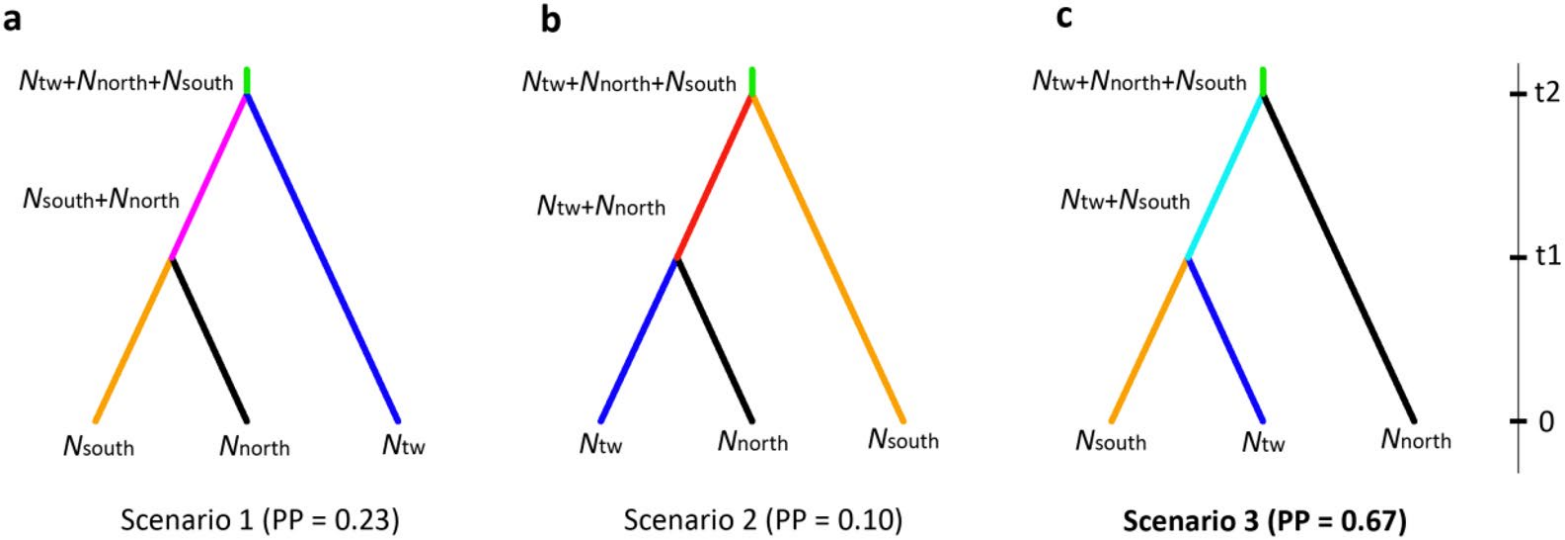
The three divergence scenarios with posteriors probability (PP) among southern, northern and Taiwan clusters of *Lindera aggregata* (**a**–**c**). The effective population size of the three clusters is labeled as *N*_south_, *N*_north_ and *N*_tw_. t1/t2, divergence times for the depicted event.

In DIYABC, scenario 3 (southern cluster and Taiwan cluster coalescing first and then coalescing with subtropical clusters) which supports the subtropical-tropical divergence receives the highest support (PP = 0.67) compared to scenario 1 (PP = 0.23, southern cluster and subtropical clusters coalescing first and then coalescing with Taiwan cluster) and 2 (PP = 0.10, Taiwan cluster and subtropical clusters coalescing first and then coalescing with southern cluster) (Fig. 2). Based on a generation time of approximate 10 years^20^, the divergence times of subtropical-tropical and Taiwan-South China populations are estimated as 154 ka (95% HPD, 64–275 ka) and 112 ka (95% HPD, 54–173 ka), respectively. Other parameter estimations are shown in Table 3 and Supplementary Fig. S4.

**Table 3:** Posterior median estimation and 95% highest posterior density interval (HPD) for demographic parameters in scenario 3 of *Lindera aggregata* in DIYABC.

	*N*_south_	*N*_tw_	*N*_north_	t1	t2	*μ*
Median	1.68 × 10^4^	5.20 × 10^3^	4.13 × 10^4^	112,000	154,000	6.17 × 10^–8^
q (0.05)	1.01 × 10^4^	2.63 × 10^3^	2.68 × 10^4^	54,000	64,000	3.81 × 10^–8^
q (0.95)	2.46 × 10^4^	8.37 × 10^3^	5.48 × 10^4^	173,000	275,000	9.13 × 10^–8^

*N*_south_, *N*_tw_ and *N*_north_ represent current population size of southern, Taiwan and northern cluster; t1/t2, divergence times in years when a generation time of 10 years is applied for the depicted event; *μ*, mutation rate per generation per locus.

**Figure 3:**
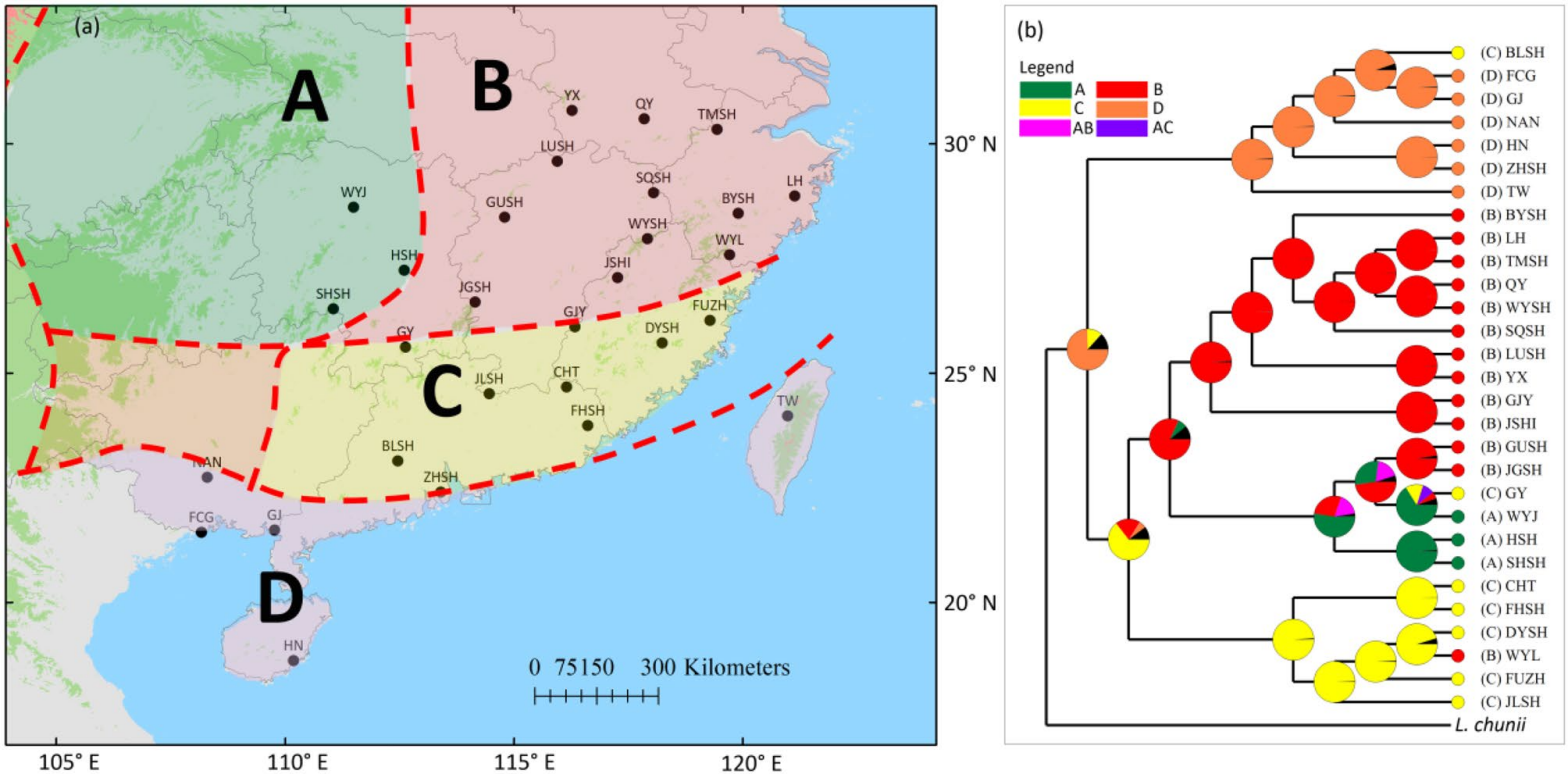
(**a**) The four major floristic divisions (A–D) in southeast China according to Wu et al.,^2^ (ArcGis 10.2, ESRI, www.esri.com), and (**b**) ancestral area reconstructions based on the Bayesian binary Markov chain Monte Carlo (BBM) method implemented in RASP using the BEAST-derived phylogeny of 29 *Lindera aggregata* populations (see Fig. 1b). Pie charts of each node illustrate the marginal probabilities for each alternative ancestral area derived from BBM, black colour indicate unknown ancestral area.

BBM analysis in RASP indicates the most possible distribution of ancestral *L. aggregata* populations is located in tropical region (D, 76%) and they experience vicariance between tropical and South China region after dispersal (D → CD → C|D, Fig. 3). The ancestral distribution of subtropical populations is likely within South China (C, 64%).

### IBD, IBE and IBR analyses

The environmental and geographic distance show weak correlation (*r* = 0.21). Based on geographic, environmental and genetic distance, Mantel test and partial Mantel test show significant IBE (*r* = 0.24, *P* = 0.03), or (*r* = 0.23, *P* = 0.04) when accounting for geographic distance, while no significant IBD (*r* = 0.11, *P* = 0.08), or (*r* = 0.07, *P* = 0.20) when accounting for environmental distance (Table 4, Supplementary Fig. S5). The multiple matrix regression with randomization (MMRR) also indicates significant IBE (*r* = 0.23, *P* = 0.04) while not IBD (*r* = 0.07, *P* = 0.38). Mantel test further shows significant IBR (*r* = 0.24, *P* = 0.03).

**Table 4:** Isolation by distance (IBD), isolation by environment (IBE) and isolation by resistance (IBR) analyses among geographic, environmental and genetic distance.

	Mantel test	Partial Mantel test	MMRR
IBD	0.11	0.07	0.07
IBE	0.24*	0.22*	0.23*
IBR	0.24*	–	–

*MMRR* multiple matrix regression with randomization.

*Indicates *P* < 0.05.

**Figure 4:**
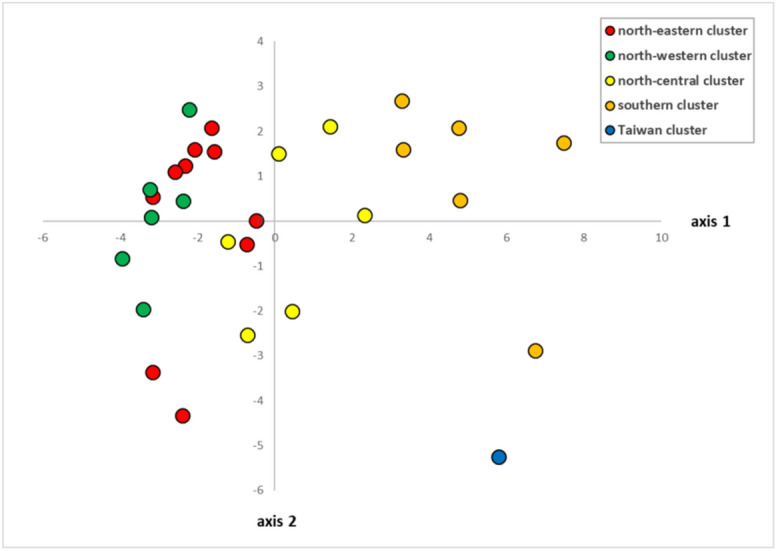
Principal component analysis (PCA) plots with 19 climatic variables (see Supplementary Table S3) of 29 *Lindera aggregata* populations. Different colours correspond to the five genetic clusters inferred by STRUCTURE analysis.

The first two axes of the principal component analysis, PCA, on climate data for the investigated populations explained 80.4% (axis 1: 57.7%, axis 2: 22.7%) of the total variation. Figure 4 and Supplementary Table S3 show that the tropical populations tend to occur in warmer areas with higher precipitations compared to subtropical populations.

## Discussion

### Late Pleistocene subtropical–tropical divergence triggered by climate

With additional 11 sampled populations, especially in the tropical and genetic admixture region^20^, more detailed genetic structure is revealed and distinct subtropical–tropical divergence is detected. Although one widespread haplotype (H3), more diverse haplotypes in southern region and no distinct structure are consistently found using chloroplast markers^20^, various analyses including genetic differentiation distribution, genetic structure, Bayesian phylogeny, ABC modeling of nuclear markers, which have much higher mutation rate^24^, successfully detect the recent subtropical–tropical divergence that can be only traced back to late Pleistocene (109.7/154 ka).

The IBD and IBE analyses indicate climatic difference rather than geographical distance is responsible for the genetic differentiation of *L. aggregata*. The PCA analysis shows that tropical populations are tend to occur in warmer areas with higher precipitations compared to subtropical populations, similar to our previous study^20^. It should be noticed that a significant IBE does not necessarily imply the occurrence of adaptation to local environments^25,26^. Short evolutionary history (since late Pleistocene) occurred in small scale compared to the late Miocene origin *Juglans* spp. that are wildly distributed in East Asia^7^. Further, the two varieties are very hard to discriminate as the difference lies only in type of pubescence, and size of leaf and flower, and intermediate types are also found during our field work. Thus, whether adaptive evolution exists between subtropical and tropical populations needs further investigations. The significance of IBR may largely due to climatic difference because the IBR implicitly conflates IBD and IBE^22^, and the resistance distance was calculated based on the ENM at present using 10 low correlated bioclimatic variables^20^.

The absence of significant IBD shows genetic barrier of Wuyi and Nanling Mountains in other species^20,27,28^ would act as dispersal corridor in *L. aggregata* as introgressions from three subtropical clusters are found^20,29^. The distinct clustering of subtropical red and green or even yellow cluster may be caused by demographic expansions. Waters et al.^30^ suggest the genetic partitioning of re-colonizing genotypes could potentially produced by a combination of occasional northward long distance dispersal (LDD) and high-density blocking. In *L. aggregata*, occasional LDD dispersed by birds^31^ combined with high-density hindering would cause genetic partitioning in subtropical region^30,32^ although low-level of genetic differentiations are found (Table 2). Taiwan Strait^33,34^ and South China Sea (SCS)^35^ should act as geographical barriers between Taiwan and the mainland populations even though no significant IBD is found. High pairwised genetic differentiations between Taiwan and other clusters combined with low genetic diversity (especially *A*_R_) in LCGs and two unique chloroplast haplotypes indicate Taiwan population is long-term isolated^36^. Bayesian phylogeny of LCGs further implies that it is likely a relict population. Thus, the Taiwan Strait and SCS have successfully impeded both pollen and seed dispersal between Taiwan and the mainland. While in Qiongzhou Strait, shared nuclear and chloroplast genetic component between Hainan island and the mainland (Fig. 1 and Supplementary Fig. S1) signify effective seed and pollen dispersal.

### Dual role of SCS and hierarchical south-north dispersal

Our previous work indicates *L. aggregata* have experienced postglacial northward range expansions from long-term persisted southern refugia populations^20^. The present study with additional populations could trace its earlier demographic history in tropical region. Dual role of SCS land bridge and hierarchical south to north dispersal shape the present distribution of *L. aggregata* populations.

In Taiwan, its cpDNA haplotypes are closely related to that from South China although limited variations are found (Supplementary Figs. S1 and S2). This closer relationship is further confirmed in ABC estimations using LCGs (Fig. 1). DIYABC estimates a similar mutation rate range (Table 2) compared to Ye et al.^20^ and a similar divergence time (112 ka) between Taiwan and South China populations with BEAST (90.7 ka). The time falls into the last interglacial when sea level raised^37^. The sea level fluctuations of SCS due to glacial-interglacial alternations can both provide dispersal corridor or barrier^38,39^. The *L. aggregata* populations likely showed continuous distribution between South China and Taiwan in early stage, while geographic isolation afterwards has resulted in genetic distinctness. Previous study on *Quercus championii* (Fagaceae) also suggests the Pleistocene SCS land bridge contributed to flora of Taiwan island by dispersal from ancestral Southwest China-Southeast Asia^35^. Some terrestrial vertebrates also display similar patterns^40^.

Hierarchical south to north dispersal of *L. aggregata* is mainly supported by the RASP analysis. *Lindera* is supposed to be tropical Asia origin as the ancestral types are concentratedly distributed in Southwest and South China^41^. Chloroplast phylogenomics further indicate that *L. aggregata* is closely related to species that are distributed in Southwest and South China^42^. As RASP indicates that the ancestral populations of *L. aggregata* are located within tropical region (Fig. 3). It can be speculated that populations in Taiwan and South China (brown and blue cluster, Fig. 1) are firstly established, likely from Southwest and/or South China. Then populations in narrow belt between Nanling-Wuyi Mt. and SCS (yellow cluster, Fig. 1) are formed from tropical populations. At last, north-most populations (red and green clusters) are colonized from southern refugia populations, likely located in South China (C) floristic sub-region as show in RASP analysis (Fig. 3), after LGM^20^.

## Conclusion

Different floristic regions in East Asia are likely result in different evolutionary histories. The present study firstly shows distinct subtropical-tropical divergence using a dominant species in the evergreen broadleaved subtropical forests in East Asia. It is an important case study supplement to the widely investigated temperate-subtropical differentiation related genetic divergence. The genetic divergence is mainly attribute to the current climatic conditions and the present distribution of *L. aggregata* populations are formed through dual role of SCS land bridge and hierarchical south to north dispersal since the late Pleistocene. In conclusion, although South-East China harbors low heterogeneity of topography and climate, subtropical-tropical divergence may also be established. However, further genetic investigations on other species are needed to verify this assumption.

## Methods

### Sampling and sequencing

We collected leaf samples of a total of 92 individuals from 11 populations (Table 1) in addition to previous 139 individuals from 19 populations^20^. There are 23 populations in Sino-Japanese Floristic Region (refer as subtropical populations) and the remaining seven populations in Paleotropical Floristic Region (refer as tropical populations)^2^ (Table 1, Fig. 1). Procedures for sample collection, DNA extraction, primer information, PCR amplification and sequencing of four cpDNA fragments and 15 LCGs loci can be found in Ye et al.^20^.

### Genetic structure

In cpDNA, DnaSP 5.10.01^43^ was used to determine haplotypes with indels treated as substitution sites. In LCGs, DnaSP was used to phase heterozygous sequences. Then, potential population structure was assessed using STRUCTURE 2.3.4^44^ with an admixture model and assuming allele frequencies to be correlated among populations. Ten independent runs were performed for each number of populations (*K*) from 1 to 10 with 100,000 Markov chain Monte Carlo (MCMC) steps of burn-in, followed by 1,000,000 steps. Ln*P*(*D*) and Δ*K* were applied to determine the most likely number of clusters. Pairwised genetic differentiation (*F*_ST_) and genetic diversity as measured by π and *A*_R_ of LCGs among potential clusters were calculated in SPADS 1.0^45^.

### Bayesian phylogeny and divergence time estimation

In LCGs, the Bayesian phylogeny of 29 populations (population WGSH was excluded due to sequencing problems) was inferred using *BEAST^46^ in BEAST 2.4^47^ with all partitions unlinked. The substitution models for all loci were the same as Ye et al.^20^. A strict clock model and Yule process with a piecewise linear and constant population size model were applied. The length of the MCMC algorithm was set to 2 × 10^9^ steps with sampling every 2 × 10^4^ steps, and the first 20% was discarded as burn-in. The estimated substitution rate of 3.4 × 10^−9^ with 95% HPD of 1.8 × 10^−9^–5.8 × 10^–9^ site^−1^ year^−1^ was applied to estimate divergence time^20^.

### DIYABC analysis

As the lineage including the Taiwan population (TW) and populations in southern cluster (NAN, FCG, GJ, HN, BLSH and ZHSH) does not receive significant support (see “Results”), the subtropical–tropical divergence was further checked through approximate Bayesian calculation (ABC) in DIYABC 2.0^48^. All individuals were subdivided into TW, southern cluster, and northern cluster (including north-western, north-eastern and north-central clusters) in subtropical region according to STRUCTURE and Bayesian phylogeny analyses (see “Results”). Three possible scenarios were simulated (Supplementary Table S2).

Each simulation was summarized by the following summary statistics: number of segregating sites, mean and variance of pairwise differences, private segregating sites, and mean and variance of number of rarest nucleotide at segregating sites with cluster, mean of segregating sites, mean of pairwise differences, and *F*_ST_ between pairs of clusters. The simulation was repeated 1 × 10^6^ times for each scenario. To compare the posterior probability of three scenarios, the 3 × 10^4^ (1%) simulated data sets closest to the observed data set were selected for the logistic regression and 300 for the direct approach. After choosing the best scenario, we estimated parameter posterior distributions taking 1 × 10^4^ (1%) simulated data sets closest to the observed data set for the local linear regression, after applying a logit transformation to the parameter values. Two independent runs were performed in all simulations.

### Ancestral area reconstructions

In order to reconstruct the geographical diversification of *L. aggregata*, the Bayesian binary MCMC (BBM) analysis implemented in RASP 3.2^49^ was performed using 80% post burn-in trees retained from the BEAST analysis of LCGs. Four geographic regions representing the current distribution were defined according to the floristic division of China^2^: A, Central China; C, South China; B, East China, and D, Tropical China. The number of maximum areas at each node was set to five. We applied 20 MCMC chains with the JC + G model running for 1 × 10^6^ generations, and sampled the posterior distribution every 100 generations with the first 10% treated as burn-in.

### IBD, IBE and IBR analyses

To infer the contributions of current geography and environment to genetic differentiation, IBD, IBE and IBR was analyzed. Pairwised genetic differentiation (*F*_ST_) of LCGs was calculated in SPADS and *F*_ST_/(1 − *F*_ST_) was used as genetic distance. The geographic distance was transformed by natural logarithm. Climate variables of the 29 sampled populations of 19 climatic variables, which was downloaded from WorldClim 1.4^51^ at a 2.5-arcmin resolution, were extracted using Spatial Analyst Tools in ArcGis 10.2 (ESRI, Redlands, CA, USA). Pairwised Euclidian distances calculated (‘dist’ function in R 3.2.3^50^) using the matrix of all 19 retrieved climatic variables was adopted to represent environmental distances. A PCA (‘princomp’ function) was performed in R^50^ on the 19 retrieved climatic variables to detect tropical-subtropical environmental differences.

The resistance-based spatial distance was calculated based on ENM at present. The predicted distribution was modeled by 10 low correlated (r < 0.8) bioclimatic variables (annual mean temperature, mean diurnal range, isothermality, temperature seasonality, maximal temperature of warmest month, mean temperature of wettest quarter, annual precipitation, precipitation of wettest month, precipitation of driest month, and precipitation seasonality) at a 2.5-arcmin resolution through maximum-entropy modelling technique (Maxent 3.3.3^52^) using 126 occurrences of *L. aggregata*^20^. The ENM resulted in a map of environmental suitability values ranging from 1 to 0 with ratio of potential distribution from high to low. Resistance values were the reverse of the suitability scores (1 − suitability), because higher suitability is expected to have lower resistance^53^. Least-cost path distances are calculated by finding the minimum total cumulative resistance between two populations^53^ using SDM Toolbox in ArcGis 10.2. ‘partial.mantel.test’ function in R^50^ was used to perform Mantel tests between genetic distance and geographic distance, environmental distance or resistance-based spatial distance, and partial mantel tests between genetic distance and geographic distance (or environmental distance) while accounting for environmental distance (or geographic distance) using 1 × 10^4^ permutations. MMRR^54^ that provides a straightforward method for estimating linear regressions among distance matrices was further used to qualify IBD and IBE. The effect of environmental distance and geographic distance, as explanatory variables, on genetic distance, as the response variable, was analyzed using the ‘MMRR’ function in R^50^ with 1 × 10^4^ permutations.

## Supplementary Information

Supplementary Information.

## Data Availability

DNA sequences are deposited in GenBank with accessions MN366253–MN366276, MN418453–MN418655.
