# Supplementary material for: Distinct late Pleistocene subtropical-tropical divergence revealed by fifteen low-copy nuclear genes in a dominant species in South-East China
Source: Sci Rep. 2021 Feb 18;11:4147. doi: 10.1038/s41598-021-83473-w (PMC7892551; doi:10.1038/s41598-021-83473-w)
Supplement: Supplementary file 1 — Supplementary Information. [file 41598_2021_83473_MOESM1_ESM.docx]

**Distinct late Pleistocene subtropical-tropical divergence revealed by fifteen low-copy nuclear genes in a dominant species in South-East China**

Jun-Wei Ye, De-Zhu Li

| **Table S1** Haplotypes derived from four chloroplast DNA fragments in *Lindera aggregata*: *trnH–psbA* (positions 1–333), *rpl16* (334–1077), *trnL–trnF* (1078–1814) and *trnS–trnG* (1815–2587). Newly derived haplotypes are labeled in bold and variable sites are labeled in red. | | | | | | | | | | | | | | | | | | | | | | | | | | | | |
| --- | --- | --- | --- | --- | --- | --- | --- | --- | --- | --- | --- | --- | --- | --- | --- | --- | --- | --- | --- | --- | --- | --- | --- | --- | --- | --- | --- | --- |
| Haplotype | n | 1 | 6 | 6 | 6 | 6 | 6 | 6 | 1 | 1 | 1 | 2 | 2 | 3 |  | 4 | 1 | 1 | 1 |  | 1 | 1 |  | 1 | 1 | 2 | 2 | 2 |
|  |  | 2 | 2 | 3 | 5 | 6 | 8 | 9 | 2 | 6 | 6 | 0 | 2 | 1 |  | 4 | 0 | 0 | 0 |  | 6 | 7 |  | 8 | 8 | 3 | 4 | 5 |
|  |  |  |  |  |  |  |  |  | 9 | 1 | 4 | 9 | 3 | 3 |  | 6 | 0 | 3 | 6 |  | 3 | 4 |  | 3 | 8 | 4 | 5 | 8 |
|  |  |  |  |  |  |  |  |  |  |  |  |  |  |  |  |  | 7 | 5 | 4 |  | 2 | 5 |  | 6 | 8 | 2 | 4 | 0 |
|  |  | C | A | A | A | G | A | C | A | C | T | G | T | I* |  | C | G | A | C |  | T | G |  | T | C | G | G | T |
| HAP1 | 8 | — | — | — | — | — | — | — | — | — | — | — | C | I |  | — | — | — | — |  | — | T |  | — | — | — | — | — |
| HAP2 | 1 | — | — | — | — | — | — | — | — | — | — | — | — | I |  | — | — | G | — |  | — | — |  | — | — | — | — | — |
| HAP3 | 168 | — | — | — | — | — | — | — | — | — | — | — | — | I |  | — | — | — | — |  | — | — |  | — | — | — | — | — |
| HAP4 | 14 | — | — | — | — | — | — | — | — | — | — | — | — | I |  | — | — | — | — |  | — | — |  | — | T | — | A | — |
| **HAP5** | 1 | — | — | — | — | — | — | — | — | — | — | — | — | I |  | — | — | — | — |  | G | — |  | — | — | — | — | — |
| **HAP6** | 4 | — | — | — | — | — | — | — | — | — | — | — | — | I |  | — | — | — | A |  | — | — |  | — | — | — | — | — |
| HAP7 | 8 | — | — | — | — | — | — | — | — | — | — | — | — | I |  | — | — | — | — |  | — | — |  | — | — | — | — | C |
| **HAP8** | 2 | — | — | — | — | — | — | — | — | — | — | — | — | I |  | — | — | — | — |  | — | — |  | — | — | A | — | — |
| HAP9 | 9 | — | — | — | — | — | — | — | G | — | — | — | — | I |  | — | — | — | — |  | — | — |  | — | — | — | — | — |
| HAP10 | 5 | — | G | T | C | T | T | T | — | — | — | — | — | — |  | — | — | — | — |  | — | — |  | — | — | — | — | — |
| HAP11 | 8 | — | — | — | — | — | — | — | — | A | — | — | C | I |  | — | T | — | — |  | — | T |  | — | — | — | — | — |
| HAP12 | 3 | — | — | — | — | — | — | — | — | — | C | — | — | I |  | — | — | — | — |  | — | — |  | — | — | — | — | — |
| HAP13 | 2 | T | — | — | — | — | — | — | — | — | — | — | — | I |  | — | — | — | — |  | — | — |  | — | — | — | — | — |
| **HAP14** | 9 | — | — | — | — | — | — | — | — | — | — | T | C | I |  | — | — | — | — |  | — | T |  | G | — | — | — | — |
| **HAP15** | 1 | — | — | — | — | — | — | — | — | — | — | T | C | I |  | — | — | — | — |  | — | T |  | — | — | — | — | — |
| **HAP16** | 2 | — | — | — | — | — | — | — | — | — | — | — | — | I |  | T | — | — | — |  | — | — |  | — | — | — | — | — |
| *Indel: GAAGGTAATAAAGA | | | | | | | | | | | | | | | | | | | | | | | | | | | | |

| **Table S2** Prior distributions for parameters used in model comparisons and command line input in DIYABC of three possible divergence scenarios of *Lindera aggregata* using 15 low-copy nuclear genes (LCGs) data. | | | | | | |
| --- | --- | --- | --- | --- | --- | --- |
| Parameters | Prior^a^ |  | Scenarios | 1 (north and south cluster coalescence first) | 2 (TW and north cluster coalescence first) | 3 (TW and south cluster coalescence first) |
| *N*_1_ | (10, 60000) |  | Command line | n1 n2 n3 0 sample 1 0 sample 2 0 sample 3  t1 merge 1 2 t1 varne 1 n1+n2 t2 merge 1 3 t2 varne 1 n1+n2+n3 | n1 n2 n3 0 sample 1 0 sample 2 0 sample 3  t1 merge 1 3 t1 varne 1 n1+n3 t2 merge 1 2 t2 varne 1 n1+n2+n3 | n1 n2 n3 0 sample 1 0 sample 2 0 sample 3  t1 merge 2 3 t1 varne 2 n2+n3 t2 merge 2 1 t2 varne 2 n1+n2+n3 |
| *N*_2_ | (10, 30000) |  |  |  |  |  |
| *N*_3_ | (10, 10000) |  |  |  |  |  |
| *μ* | (1×10^-8^, 1×10^-7^)^b^ |  |  |  |  |  |
| *t1* | (0, 20000)^c^ |  |  |  |  |  |
| *t2* | (0, 30000) |  |  |  |  |  |
| ^a^All priors were uniformly distributed. The unit of timing is generation; ^b^HKY substitution model was used, ^c^divergence time in generations. n1, n2, and n3 represent cluster of north, south and TW, respectively. | | | | | | |

| **Table S3** Bioclimatic variables and standardized loadings for the two first axes of the principle component analysis (PCA) of 29 populations of *Lindera aggregata*. | | | |
| --- | --- | --- | --- |
| Variable | Description | First axis (PC1) | Second axis (PC2) |
| Bio1 | Annual mean temperature | 0.278 | 0.160 |
| Bio2 | Mean diurnal range (mean of monthly (max temp - min temp)) | -0.092 | 0.101 |
| Bio3 | Isothermality (BIO2/BIO7) (× 100) | 0.269 | 0.065 |
| Bio4 | Temperature seasonality (SD × 100) | -0.277 | 0.101 |
| Bio5 | Max temperature of warmest month | 0.039 | 0.393 |
| Bio6 | Min temperature of coldest month | 0.294 | 0.057 |
| Bio7 | Temperature Annual Range (BIO5-BIO6) | -0.277 | 0.105 |
| Bio8 | Mean temperature of wettest quarter | 0.206 | 0.243 |
| Bio9 | Mean temperature of driest quarter | 0.284 | 0.076 |
| Bio10 | Mean temperature of warmest quarter | 0.134 | 0.369 |
| Bio11 | Mean temperature of coldest quarter | 0.294 | 0.062 |
| Bio12 | Annual precipitation | 0.103 | 0.423 |
| Bio13 | Precipitation of wettest month | 0.178 | 0.345 |
| Bio14 | Precipitation of driest month | -0.265 | 0.119 |
| Bio15 | Precipitation seasonality (coefficient of variation) | 0.281 | 0.090 |
| Bio16 | Precipitation of wettest quarter | 0.178 | 0.340 |
| Bio17 | Precipitation of driest quarter | -0.265 | 0.145 |
| Bio18 | Precipitation of warmest quarter | 0.191 | 0.280 |
| Bio19 | Precipitation of coldest quarter | -0.207 | 0.202 |


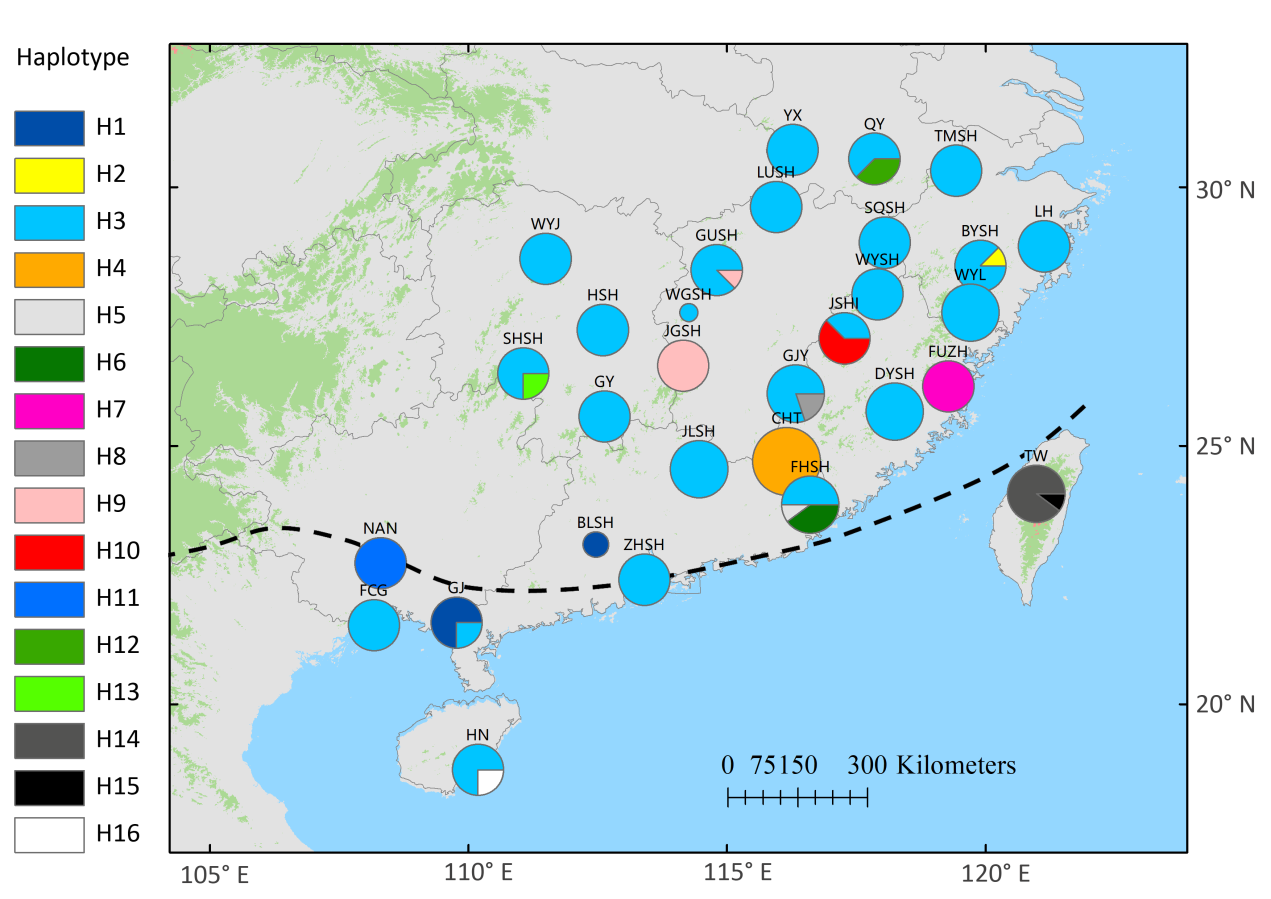


**Figure S1** Geographical distribution of the 16 identified *Lindera aggregata* chloroplast DNA haplotypes (ArcGis 10.2, ESRI, www.esri.com). All circle sizes are proportional to sample sizes. The dashed black line represents the boundary of northern subtropical and southern tropical floristic regions.


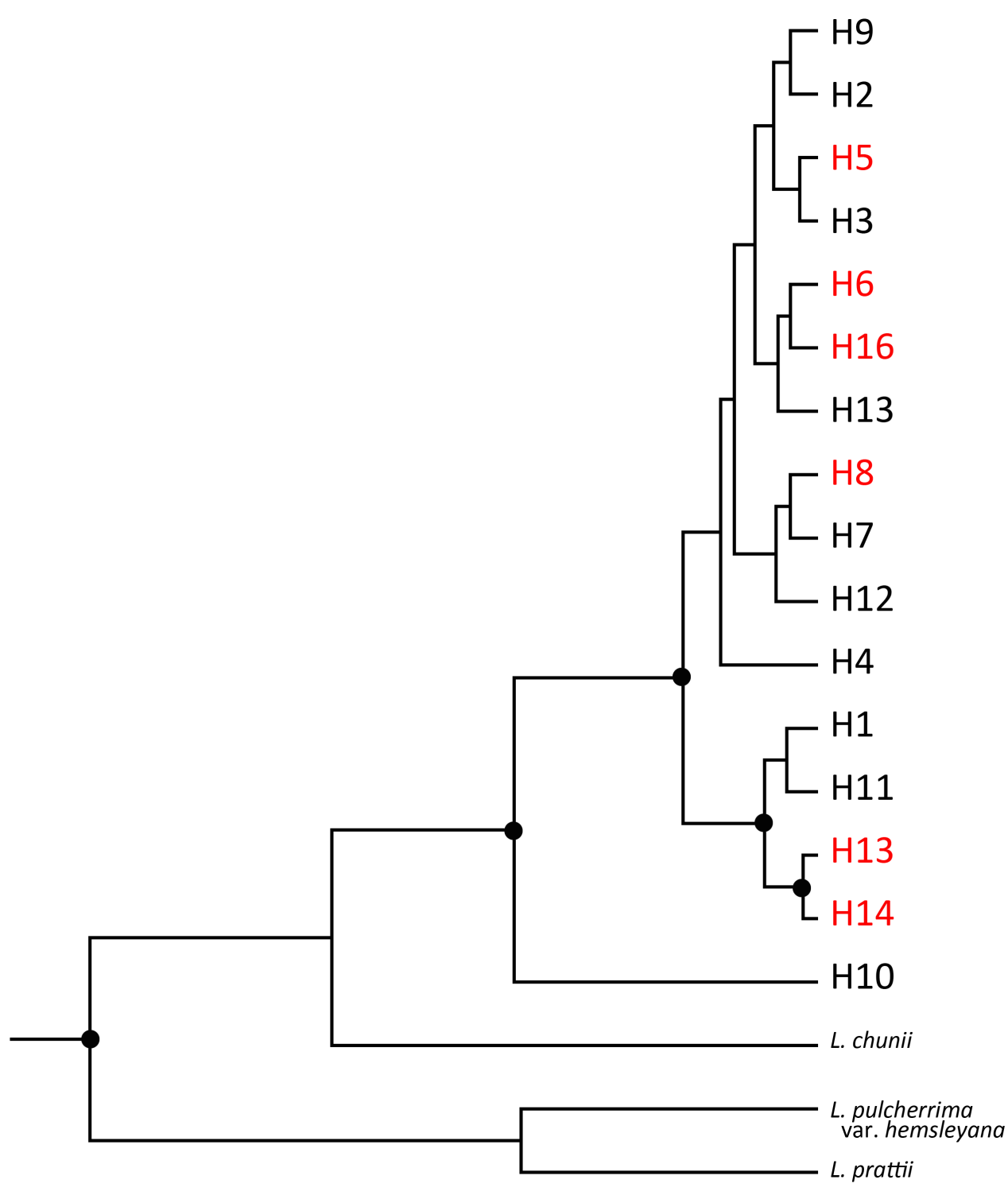


**Figure S2** BEAST-derived chronogram with statistically significant posterior probabilities (pp > 0.95) labeled with black dots. The newly derived haplotypes are label in red.


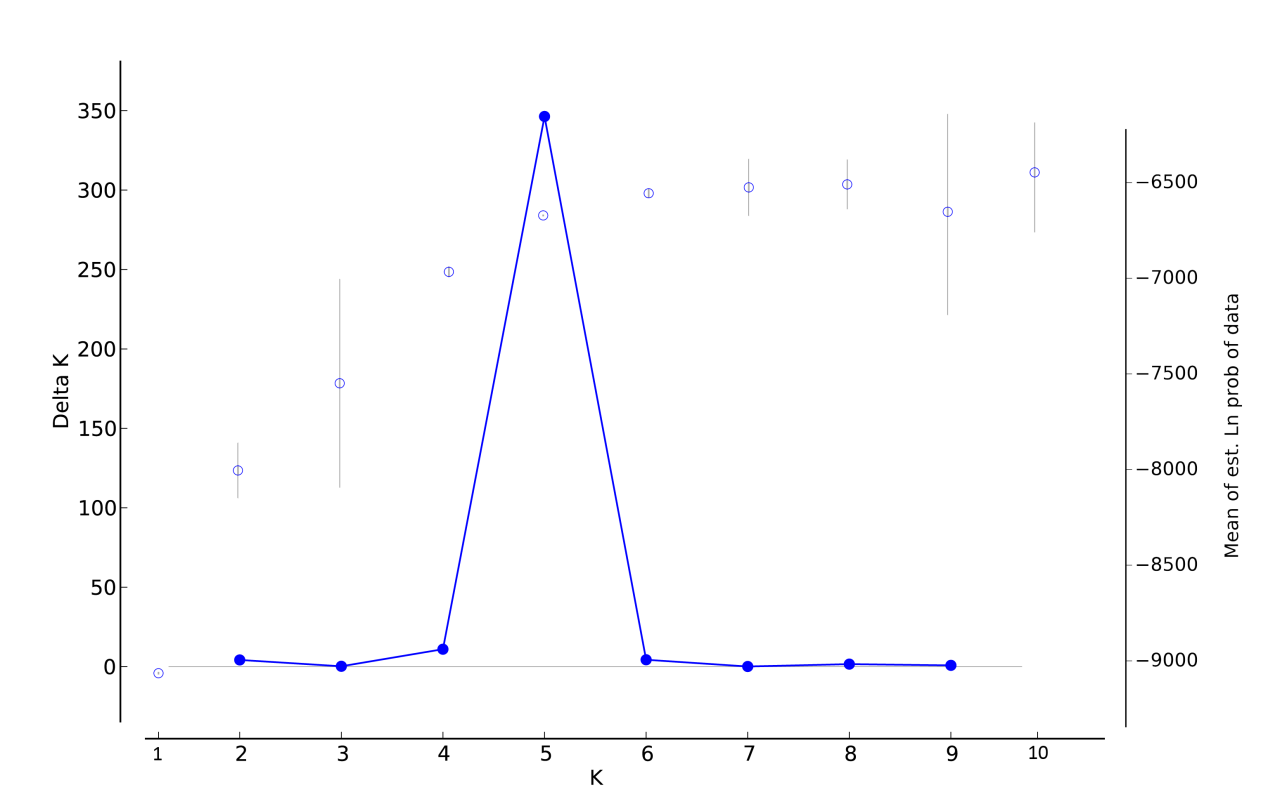


**Figure S3** Delta-*K* and Ln*P(D)* values from the STRUCTURE analysis on 29 *Lindera aggregata* populations with predefined group number K = 1–10. Standard deviations of Ln*P(D)* obtained from 10 independent runs for each group number are also shown.


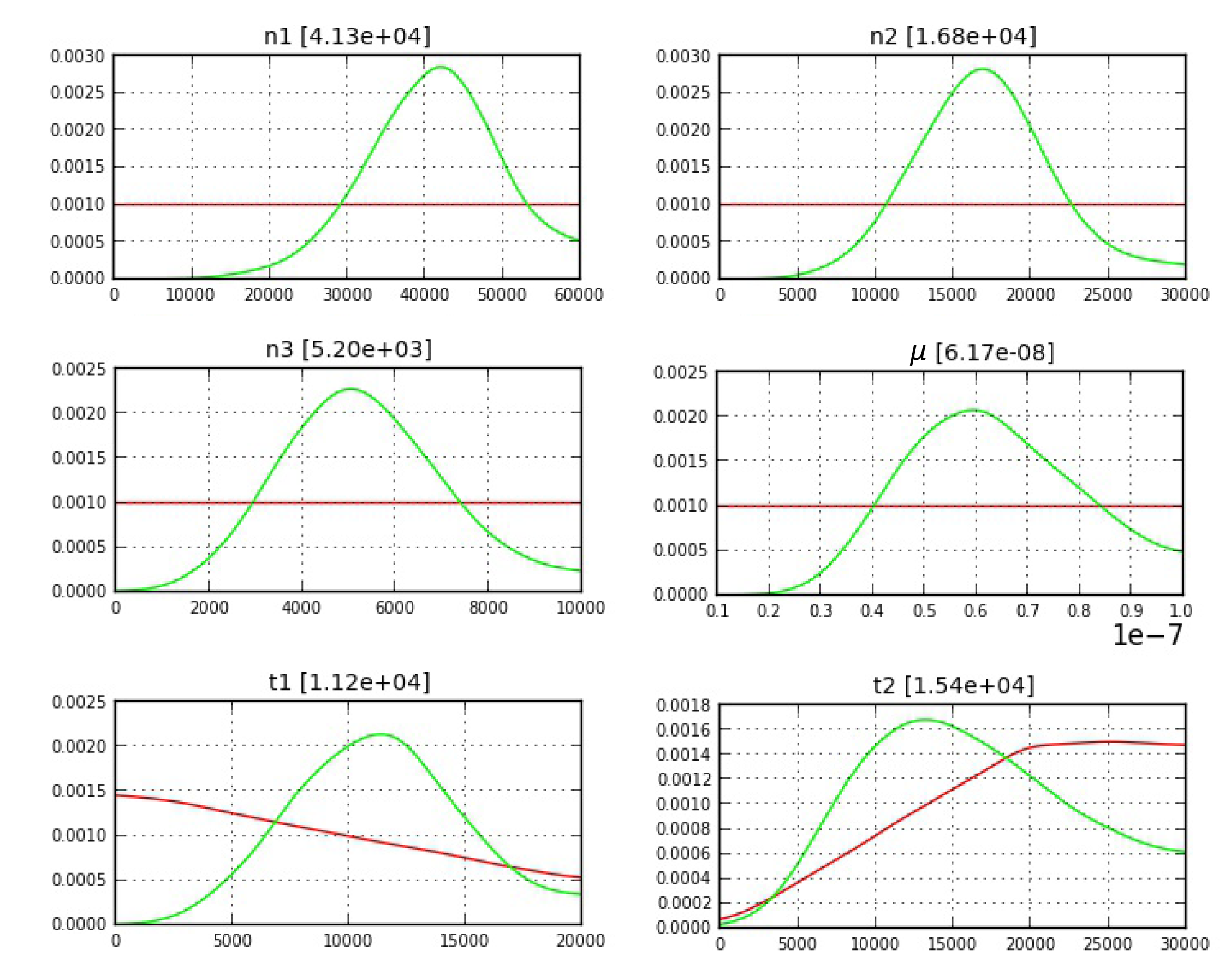


**Figure S4** Prior (red line) and posterior (green line) distribution of parameter estimations in Scenario 3 in DIYABC. n1, n2, and n3 represent cluster of north, south and TW, respectively; μ, mutation rate per generation per locus; t1/t2, divergence times in generations for the depicted event.


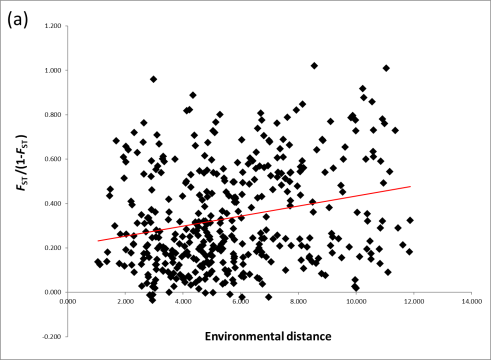


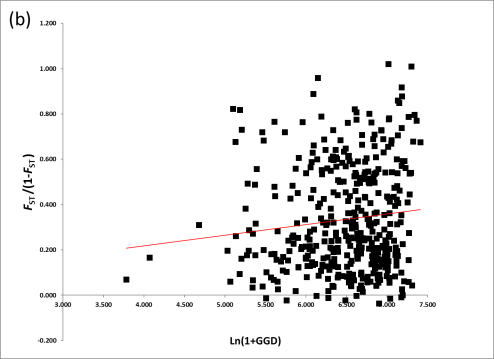


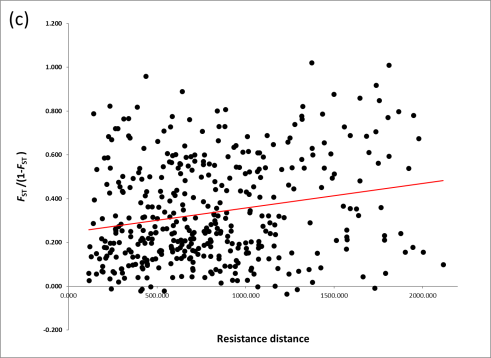


**Figure S5** Correlations between genetic differentiation with environmental distance (a), geographic distance (b) and resistance distance (c). Red line represents the trend line.
